# Understanding why resident doctors leave the NHS and what can be done to retain them: protocol for a realist synthesis

**DOI:** 10.1136/bmjopen-2025-107410

**Published:** 2025-08-06

**Authors:** Naomi Klepacz, Anna Melvin, Simon Briscoe, Daniele Carrieri, Florence Katie Lock, Priya Patel, Kevin Teoh, Geoff Wong, Karen Mattick

**Affiliations:** 1 Medical School, University of Exeter, Exeter, UK; 2 Department of Public Health and Sport Sciences, University of Exeter, Exeter, UK; 3 University Hospitals Dorset NHS Foundation Trust, Poole, England, UK; 4 Department of Organizational Psychology, Birkbeck University of London, London, Greater London, UK; 5 Nuffield Department of Primary Care Health Sciences, Oxford University, Oxford, UK

**Keywords:** Health Workforce, Physicians, MEDICAL EDUCATION & TRAINING, Human resource management, Job Satisfaction

## Abstract

**Abstract:**

**Introduction:**

The UK’s medical workforce is under increasing strain, and this is compounded by increasing numbers of resident doctors diverging from specialist training pathways, instead entering non-training roles, reducing clinical hours or leaving the profession or UK workforce entirely. These decisions are shaped by both individual motivations and wider structural conditions, including unsatisfactory working conditions, limited flexibility and a perceived lack of support or autonomy. While pursuing alternative career routes offers personal and professional benefits, they can also delay progression to senior clinical roles, contributing to workforce instability. There remains limited understanding of how best to support retention, particularly given the varied contexts, settings and career trajectories of resident doctors. This realist synthesis will examine how, why and in what contexts resident doctors leave the National Health Service, and what interventions might support their retention.

**Methods and analysis:**

This realist synthesis will follow Realist And Meta-narrative Evidence Synthesis: Evolving Standards guidance and will be conducted in five iterative steps: (1) identifying existing theories to develop an initial programme theory; (2) undertaking formal and purposive searches to identify relevant UK-based literature; (3) selecting documents based on relevance and rigour; (4) extracting and coding data to support the development of explanatory insights; and (5) synthesising findings using a realist logic of analysis to develop and refine context-mechanism-outcome configurations. An advisory group will guide the review throughout. The final programme theory will inform the development of evidence-based recommendations and design principles to support resident doctor retention.

**Ethics and dissemination:**

Ethical approval is not required for this synthesis of existing literature. Findings will be disseminated through academic publications, conference presentations and accessible formats, including infographics, plain English summaries and blog posts. Target audiences include resident doctors, medical educators, workforce planners and policymakers.

**Study registration:**

PROSPERO, CRD420251004453.

**STRENGTHS AND LIMITATIONS OF THIS STUDY**

- Stakeholder engagement will ensure relevant literature is identified and meaningfully interpreted, strengthening the development of the programme theory.
- As much of the evidence on medical workforce retention exists in policy and organisational literature, incorporating grey literature alongside peer-reviewed studies ensures a comprehensive evidence base.
- The research team brings a diverse range of expertise, including clinical experience, realist methodology and information science, which will support a rigorous and well-rounded synthesis.
- Any paucity of specific research on the retention of resident doctors in the National Health Service may limit the depth of understanding developed.

## Introduction

 Career decisions made by resident doctors are attracting increased attention amid concerns over the long-term supply of senior clinicians in the UK’s National Health Service (NHS).^1^ Resident doctors—previously known as junior doctors—are qualified doctors below the level of consultant or general practitioner (GP).^2 3^ They form the largest group in the UK’s medical workforce.^4^ While many within this group follow specialist training pathways, increasing numbers are entering locally employed (LE) roles (eg, trust grade doctors, clinical fellows), taking career breaks, reducing their hours or leaving the NHS.^4^ These choices, while reflecting individual needs or preferences, also signal a broader shift away from the established pipeline for developing a skilled and sustained consultant and GP workforce. Understanding the context and causal mechanisms shaping early medical career decision-making, and the potential interventions that may support the timely progression to senior clinical roles, is essential if the NHS is to meet the rising demand for care.

In the UK, the postgraduate medical training pathway typically progresses from the 2-year Foundation Programme (FY1-FY2) into core or speciality training of 3–8 years duration, culminating in a certificate of completion of training (CCT), which grants entry onto the specialist or GP register.^5^ This structured and standardised pathway, introduced under the modernising medical careers^6^ reform, was designed to streamline progression and reduce the time taken to become a GP or consultant. However, these reforms have been criticised for creating rigid, high-pressure systems that push early-career doctors to make specialty decisions before they feel ready and offering limited opportunities for broad experience or exploration.^7^,^9^ Many resident doctors report facing burnout, heavy workloads, a loss of autonomy, a lack of appropriate training places and insufficient flexibility within training.^10 11^ A growing proportion are choosing to take a career break, work abroad, take an academic post or enter LE roles within the NHS after completing their foundation training rather than moving directly onto a core or speciality training programme. Others choose to pursue a portfolio pathway, which provides greater autonomy and flexibility while allowing them to obtain the CCT required for entry onto the specialist or GP register.^4^

While alternatives to specialist training pathways can offer significant individual benefits—including enhanced skills, diverse clinical experience and time for personal or professional development^12^—they typically also lengthen the time taken to reach consultant or GP level, slowing the replenishment of the senior medical workforce. At the same time, healthcare demand is rising sharply as the UK population grows, ages and presents with more complex health needs. To meet projected demand, hospital activity will need to increase by nearly 40% over the next 15 years.^1^ Yet 24% of the consultant workforce are over the age of 55 years, with many currently retiring around the age of 61 years, earlier than the UK average retirement age.^13^ Similarly, although the number of GP training places has increased over the last decade, this has been more than offset by the number of GPs retiring early or working part-time.^14^ Indeed, it is estimated that, on average, nearly two training posts are required to get one fully qualified, full-time equivalent GP joiner because the proportion of GPs working full-time has fallen.^15^ It is estimated that the number of GPs will, therefore, need to increase by 46% by 2036/7 to meet the projected patient care demand.^14 16^ If increasing numbers of resident doctors continue to pursue alternative routes, the NHS risks not only further shortages but also a lost return on the substantial public investment in training, estimated at £392 469 per doctor up to FY2 and £659 509 to the consultant level.^17^

Despite widespread recognition of the issue, the mechanisms driving resident doctor retention (or lack thereof) are not yet fully understood. A recent integrative review by Lock and Carrieri^18^ highlighted a combination of push factors, such as poor working conditions, inadequate supervision, emotional exhaustion and feeling undervalued, and pull factors, including the appeal of greater autonomy, improved work-life balance and opportunities for personal or professional growth. Similar forces have been identified in the decision to take a post-foundation years training break—the most common point at which to take a career break and a natural breaking point in the training pathway.^19^ Church *et al*^8 19^ describe how the pressures of burnout, high workloads, rigid training structures, unsupportive cultures and the attraction of greater flexibility, autonomy and time to explore other opportunities contribute to this decision. For some doctors, taking a break at this point in their career was framed as a positive opportunity for exploration and growth; for others, it represented a reluctant response to a system that failed to meet their needs.^19^

Understanding and addressing tensions between the NHS’s need for a sustainable senior clinical workforce and the push and pull factors shaping early-career decisions is therefore essential. While factors such as burnout, workload and rigid pathways can drive doctors away from core or speciality training, alternative routes may offer valuable opportunities for skill-building, reflection and personal growth that, if properly recognised and supported, could strengthen long-term retention and engagement across the career span.^4^ The General Medical Council (GMC) has called for the development of more flexible professional pathways that account for the skills and experience gained outside formal training.^4^ Evidence from doctors who left NHS training to pursue careers overseas suggested that more supportive and flexible systems can restore well-being and professional motivation, highlighting the potential value of well-structured alternatives to the current training pathways.^20^ Realist research has proved well placed to explore the importance of addressing organisational and systemic factors, showing that poorly aligned or tokenistic interventions can inadvertently worsen morale, reduce trust and increase intentions to leave.^21 22^ Emerging evidence on organisational interventions reinforces that targeting structural factors—such as rota design and staffing levels—can meaningfully enhance well-being and retention, with initiatives like annualised hours, bespoke rostering and structured peer support already showing promise in reducing resident doctor turnover.^23^

However, much of the evidence is fragmented, with studies often focused on specific interventions, settings or specialties. As a result, there is limited understanding of how these mechanisms operate across contexts, or how they can be leveraged to inform targeted and scalable strategies for improving retention. Plans to increase workforce numbers will only succeed if existing staff are retained and supported to progress into senior roles. Therefore, it is essential to understand why resident doctors leave the NHS and what can be done to retain them. Findings will inform future workforce policy and help strengthen the retention and development of the NHS’s clinical workforce.

### Aims and objectives

This review aims to improve understanding of how, why and in what contexts resident doctors leave the NHS and what can be done to retain them. Using this knowledge, we will develop recommendations and design principles for interventions to support retention strategy efforts by NHS employers, healthcare leaders and policymakers.

### Objectives

Conduct a realist review of existing literature to develop and test realist programme theories on resident doctor retention, informed by advisory group consultation, literature review and iterative realist analysis.

Supported by our advisory group, we use the realist programme theories to develop recommendations and design principles for interventions aimed at improving the retention of resident doctors for employers, leaders and policymakers.

### Research questions

1. In which contexts do some (and not other) resident doctors leave the NHS and why?
2. To what extent and how do the causal explanations/mechanisms differ (eg, across individuals, groups, specialities, settings and timepoints)?
3. What interventions to retain resident doctors are described in the published literature?
4. When, why and how are these interventions more likely to work?

## Methods and analysis

This study employs a realist synthesis, a form of theory-driven evidence synthesis, to investigate the complex factors influencing the retention of resident doctors. Multiple interacting causes shape retention outcomes, and a realist synthesis is uniquely suited to understanding these complex interactions between contexts and mechanisms (causal processes) and how they generate specific outcomes. Rather than merely describing the issue, this approach seeks to explain how and why context can influence outcomes. The research team has extensive experience conducting realist syntheses and will be supported by an advisory group, ensuring that the research remains grounded in real-world perspectives and experiences from the outset and throughout the study. Through iterative cycles of theory building and testing, a realist programme theory will be developed and tested (confirmed, refuted or refined) to provide an explanation of why resident doctors leave the NHS and how they can be retained. This review will follow Pawson’s five iterative steps for realist synthesis^24^ and the Realist And Meta-narrative Evidence Syntheses-Evolving Standards (RAMESES) quality and publication standards for realist syntheses.^25^ The search will be run in the summer of 2025 and analysis will continue into 2026.

### Patient and public involvement

We will work closely with an advisory group throughout the review. The group will include resident doctors with a range of career experiences and backgrounds—including those who have left the NHS and those who have remained—alongside patient and public contributors, medical educators, employers and national stakeholders (eg, NHS England, Royal Colleges, GMC). The advisory group will support the development of the programme theory, help identify relevant literature and co-develop evidence-informed recommendations that are meaningful to policy and practice. Four online meetings will take place over the course of the review, with continued communication between meetings to support ongoing, iterative engagement.

### Definitions and terminology

Recognising that career decisions are rarely binary, this review takes a broad view of what it means to be retained within the NHS. The term ‘*retention*’ refers to the continuation of employment as a resident doctor within the NHS in some capacity. While some doctors may leave the NHS prematurely and permanently (eg, emigrating or changing careers), others leave only temporarily (eg, working abroad for a year) before returning to work in the NHS. Doctors can be retained in a variety of formats. They might choose to take short career breaks (eg, working as a clinical fellow for a year after foundation training), diversify their careers with additional roles or training (eg, in medical education or research), work less-than-full-time and/or work locally rather than applying for a national training programme. This review will consider both actual and intentions to stay or leave.

Terminology in the UK is evolving. The term ‘*resident doctors’* is now used in place of ‘*junior doctors’*,^2^ though usage remains inconsistent across contexts. In this review, ‘*resident doctors’* refers to all qualified doctors below the level of GP or consultant; that is, doctors who have not yet obtained the CCT required for entry onto the GP or specialist register. To understand who leaves the NHS, why and in what context, this review includes doctors who are outside of clinical training, such as those in LE roles, working abroad, on career breaks, in academic posts, employed in non-NHS settings or who have left the profession altogether. Although ‘*specialist, associate specialist and specialty (SAS) doctors*’ are not typically classified as resident doctors, we will include them too, to reflect the broader workforce context and since many of the issues are shared. Understanding who, why and in what context doctors elect to enter SAS roles is important for understanding retention.

The term ‘*intervention*’ refers to any action, strategy, programme or policy aimed at supporting resident doctors to remain in, or return to, NHS employment. Interventions may be formal (eg, with a defined structure or delivery mechanism) or informal (eg, staff-led, emergent or ad hoc) or a combination of both. They may be discrete or combined (such as within a programme).^26^ They may occur at any system level:

- Macro (eg, national policy, regulatory or educational bodies)
- Meso (eg, NHS trusts, postgraduate training bodies, employing organisations)
- Micro (eg, individual or team-level support)

### Step 1: locating existing theories

An initial programme theory (IPT) will be developed as a foundation for the review, offering preliminary ideas on why resident doctors choose a particular career path and how this might vary across different groups. The first step will involve identifying existing theories that explain the factors influencing doctor retention and the interventions designed to improve it, using these insights to shape the IPT. This will be achieved through: (a) consultation with the advisory group, who will contribute insights based on their experiences; (b) drawing on previous research, which has identified key causal factors influencing retention, such as connection, well-being and working conditions—factors that could inform intervention design^1021^,^23 27^ and (c) exploratory literature searches, incorporating empirical findings and existing formal theory.^28^ These will include informal searches of bibliographic databases (eg, MEDLINE), forward and backward citation searches and reviews of grey literature from relevant medical organisations and charities to identify unpublished research, such as surveys. The IPT will be refined through iterative discussion with the research team, synthesising insights from these different sources. Once developed, the IPT will be presented to the advisory group for feedback and further refinement before formal testing with evidence begins.

### Step 2: searching for evidence

*Formal search:* the second step will involve conducting a formal literature search to identify relevant papers for inclusion in the review. This search will be informed by the IPT from Step 1 and will be designed, piloted and conducted by an information specialist (SB). The IPT will also guide the choice of databases, but we anticipate searching Ovid MEDLINE, Embase and PsycINFO. As is common in realist syntheses, we will consider resident doctors’ experiences across different specialties and settings, allowing for meaningful analysis of the factors influencing retention (see Step 3). We expect to use search terms such as ‘junior doctor’, ‘postgraduate doctor’, ‘foundation doctor’, ‘trainee doctor’, ‘retention’, ‘attrition’, ‘turnover’ and ‘intention to leave’. Subject headings relevant to each database will also be used (eg, Medical Subject Headings for Ovid MEDLINE). However, the exact terms, syntax and search terms will be refined based on the results of Step 1, discussion with our information specialist and pilot testing. We will apply a date cut-off of 2005, capturing literature from the past 20 years. This reflects the introduction of the modernising medical careers pathway, which marked a major shift in postgraduate medical training in the UK; studies predating this reform are unlikely to reflect the current context of doctor retention.

*Grey literature:* relevant grey literature will be identified through a structured search of the Health Management Information Consortium database alongside the formal literature search. Targeted searches using the terms ‘doctor’ and ‘retention’ will also be conducted on the websites of key national organisations involved in medical workforce policy, research and regulation (eg, GMC, British Medical Association, NHS England, UK Foundation Programme). We will also review the bibliographies of all included documents and seek recommendations for relevant papers through the advisory group membership and review team members.

*Additional searching:* realist syntheses may involve additional searching to support the ongoing development and testing of programme theories. As new areas of interest emerge, targeted searches will be designed, piloted and conducted in collaboration with our information specialist to identify relevant literature. For example, supplementary searches may be undertaken to explore topics such as organisational culture, well-being or training environments,^29^ helping to confirm, refute or refine specific aspects of the developing programme theory by expanding the breadth and depth of evidence available to us. Where needed, we will use search strategies tailored to identifying theory-informing data.^29^ Literature identified through additional searching will be screened and appraised by the research team, with inclusion and exclusion criteria developed through team discussion and aligned with realist principles. In keeping with the methodology, a broad range of sources will be considered, including intervention studies, qualitative research, grey literature, commentaries and policy reports.

### Step 3: document selection

Documents will be appraised against predefined inclusion criteria through a two-stage screening process: title and abstract screening, followed by full text screening.

*Title/abstract screening:* the reviewers will conduct an initial screen of all citations, applying the inclusion and exclusion criteria (table 1) to titles and abstracts in Covidence.^30^ One reviewer will screen each citation and, to ensure consistency and rigour, a minimum 10% random subsample of all retrieved citations will be independently screened by a second reviewer. Any discrepancies will be resolved through discussion; unresolved disagreements will be referred to the full project team for adjudication by majority vote.

**Table 1:** Inclusion and exclusion criteria

Inclusion criteria	Exclusion criteria
English language. Relates to resident doctors (any doctor who has graduated from medical school and is below consultant or GP level), also including SAS doctors. Relates to retention (as broadly conceptualised in this study). For international literature (outside of the UK), only sources that are directly relevant and appear to offer rich theoretical insights will be included.	Consultant or GP focused only (without including resident doctors). If it includes consultants/GPs and resident doctors, it will be excluded where it is not possible to differentiate the findings from resident doctors. Medical student focused only. If it includes medical students and resident doctors, it will be excluded where it is not possible to differentiate the findings from resident doctors. Other professions. If it includes other professions and resident doctors, it will be excluded where it is not possible to differentiate the findings from resident doctors.

GP, general practitioner; SAS, specialist, associate specialist and specialty.

*Full text screening:* full text documents will be selected for inclusion in the review by considering their relevance and rigour. Relevance refers to whether the data can contribute to theory building or testing, while rigour concerns whether the methods used to generate that data are credible and trustworthy.^28^ In line with the principles of realist synthesis, inclusion will not be determined by the overall methodological quality of a study, as valuable ‘nuggets’ of data may be found even in lower-quality studies.^31^ As an example of how we will operationalise rigour, if a document includes data from a questionnaire, we will consider this more trustworthy if the questionnaire has published reliability and validity data.

As with the title and abstract screening, each document will be screened by one researcher in Covidence^30^ and, to ensure consistency and rigour, a minimum 10% random subsample of all retrieved citations will be independently screened by a second reviewer. Full text screening will start with UK literature before moving onto international literature.

Based on the appraisal for relevance and rigour, included documents will be categorised as making either major or minor contributions to answering the research questions. This approach will allow us to focus greater analytical attention on documents making major contributions, while still retaining useful insights from the broader literature. Our provisional criteria for classifying documents are:

#### Major

- Documents that contribute relevant data to the research questions and are conducted in an NHS setting.
- Documents that contribute relevant data to the research questions and are judged to clearly help to identify mechanisms that could plausibly operate in the NHS.

#### Minor

- Documents where resident doctors form a small proportion of the population studied.
- Documents conducted in healthcare systems markedly different to the NHS (eg, private healthcare), but where the mechanisms identified could plausibly operate in the context of NHS resident doctor retention.

These criteria will be informed by Step 1 and discussed within the team during the review to ensure shared understanding and consistency. A minimum 10% random subsample of included documents will be assessed independently by a second reviewer, and their classification discussed. Any disagreements will be resolved through discussion, and unresolved issues will be reviewed by the wider project team. Title/abstract and full text screening will be conducted in Covidence.^30^

### Step 4: extraction and organisation of evidence

Initial explanatory insights will be noted alongside the appraisal process using a Word template, as the depth of insight offered by a document often relates to its relevance and classification as major and minor. Formal data extraction will involve reading the full texts of included documents and coding explanatory insights relating to the IPT within the PDF and then extracting these to an Excel and/or Word file with accompanying details and analytical notes. Coding will be both deductive (informed by the IPT) and inductive (drawing on new insights arising from the literature). Each new insight will contribute to the refinement of the programme theory. As the IPT is refined, previously analysed documents may be revisited to search for data relevant to newly emerging components of the theory. In parallel, we will extract document characteristics into a separate Excel spreadsheet to provide a descriptive overview of the included literature.

Coding will begin with documents classified as making a major contribution to the research questions. Documents classified as minor will be drawn on as needed to address specific areas of the programme theory. The aim is not to exhaustively analyse all available literature but to reach theoretical saturation in our understanding of resident doctor retention and the mechanisms that support it, that is, gather sufficient evidence to “*satisfy the theoretical need or answer the question*.”^32^ As our causal explanation evolves, documents may be reclassified from major to minor (or vice versa), with all changes documented to ensure transparency and maintain a clear audit trail. For example, if theoretical saturation is reached using the UK literature, then international literature will not be included, as it is less relevant to the study focus on the NHS.

Coding will be completed by one reviewer for each source. But to ensure rigour and support interpretive reflexivity, a minimum 10% random subsample of documents will be independently reviewed by a second reviewer at each of the above stages. These will be discussed with the wider team to promote consistency and explore alternative interpretations.^33^

### Step 5: synthesis and interpretation of evidence

We will synthesise the extracted data using a realist logic of analysis to identify and refine context-mechanism-outcome configurations (CMOcs) that explain why resident doctors leave the NHS, and what interventions may support their retention across different contexts and settings.^28^ This approach will allow us to explore, for example, why a retention intervention may be effective in one specialty or setting, but not another, and how different contextual factors influence those outcomes.

A combination of synthesis techniques will be used, including concept mapping and iterative team-based analysis. Interpretive insights will be discussed across the research team and with our advisory group to refine the programme theory and ensure relevance to practice and policy.

Realist synthesis is particularly well-suited to addressing complex questions by identifying underlying mechanisms that operate across varying contexts. Examining studies from different specialties and settings will enable the identification of transferable principles. Given the evolving backdrop of NHS workforce challenges, including the impact of the COVID-19 pandemic, we anticipate that comparisons of pre-pandemic and post-pandemic experiences and interventions may offer further insights into how context shapes mechanisms and outcomes.

We will move iteratively between analysing specific examples from the literature, refining the programme theory, and conducting targeted additional searches to test emerging theoretical components. The synthesis will use interpretive cross-case comparison to understand how and why observed outcomes occur.

In line with common practice in realist reviews,^34^ we will apply the following analytic strategies:

- Juxtaposition of data sources: where data from one source helps illuminate findings from another.
- Reconciliation of conflicting data: to understand why apparently similar contexts yield different outcomes.
- Adjudication of data: based on the methodological strengths or weaknesses of individual sources.
- Consolidation of data: to explain patterns in outcomes across varying contexts.

Through this process, we will refine a theory of what works to retain resident doctors, for whom, in what circumstances and why. The final programme theory will be reviewed by the advisory group to inform practical, evidence-based recommendations for policy and workforce planning.

## Ethics and dissemination

As this is a realist synthesis of existing literature, no formal ethical approval is required. The research team will observe good research practice throughout and follow the RAMESES guidelines^35 36^ for conducting and reporting the review. The final synthesis is expected to generate CMOcs that help explain why resident doctors leave the NHS and what might support their retention. These findings will inform evidence-based recommendations relevant to both policy and practice.

We will draw on the networks and expertise of the research team, advisory group and wider collaborators to ensure that findings are disseminated appropriately and with maximum impact. Outputs will include peer-reviewed publications and conference presentations, as well as accessible formats such as plain English summaries, infographics and podcasts. Key audiences will include resident doctors; postgraduate educators, supervisors and training programme directors; NHS employers and workforce planners; and national stakeholders involved in workforce policy and medical education (eg, NHS England, the GMC, Royal Colleges, the UK Foundation Programme Office, Association for the Study of Medical Education and the Academy of Medical Educators). Patient and public contributors will also be engaged through tailored communication strategies. The advisory group will support the development of dissemination materials to ensure they are clear, relevant and accessible across clinical, educational and policy settings.
